# Experimental Optical Testing and Numerical Verification by CuFSM of Compression Columns with Modified Channel Sections

**DOI:** 10.3390/ma14051271

**Published:** 2021-03-07

**Authors:** Piotr Paczos, Aleksandra M. Pawlak

**Affiliations:** Faculty of Mechanical Engineering, Institute of Applied Mechanics, Poznan University of Technology, 5 Marii Skłodowskiej-Curie Str., 60-965 Poznań, Poland; piotr.paczos@put.poznan.pl

**Keywords:** thin-walled column, compression, critical load, limit load, experimental analysis, numerical analysis

## Abstract

Thin-walled channel columns with non-standard cross-section shapes loaded with gradually increasing compressive force applied at the geometric centre of gravity of the cross-section were the subject of the investigations presented in this paper. The aim of the research was to determine which of the columns has the most favourable geometrical characteristics in terms of the applied load. The main investigation was an experimental study carried out using two methods: strain gauging and the optical method. Based on strain gauging, the critical forces were determined using the strain averaging method and the linear regression tangent to compression plot method. In addition, modern optical tests were performed using the ARAMIS system. The buckling forces at which the first signs of buckling appear and the buckling modes of columns were determined. The results obtained from the experimental tests were used to validate the results of numerical tests carried out using the Finite Strip Method (CuFSM). Based on this method, the values of critical forces and the percentage contribution of individual buckling forms to the loss of stability of the compressed columns were determined.

## 1. Introduction

Thin-walled structures made by cold forming are becoming increasingly popular due to their high strength, simple manufacturing technology and ease of assembly while remaining lightweight. The ability of thin-walled structures to carry relatively high loads often depends not on their strength, but on their resistance to loss of stability, which is difficult to assess. In the design of such structures, special attention must be paid to the limitations associated with local, general and distortional loss of stability. Due to these limitations, testing and analysis of thin-walled structures should not be limited to a single method.

This paper presents an experimental and numerical study in which four columns with modified cross-section shapes were analysed. The columns were loaded with a gradually increasing compressive force applied at the geometric centre of gravity of the cross-section. The aim of the study was to determine which of the analysed columns has the most favourable geometrical characteristics in terms of the applied force.

The most commonly used methods for measuring deformation of various types of structures are strain gauge methods. Banat et al. [1] analysed the buckling and post-buckling behaviour of columns loaded with axial compressive force. They performed experimental studies by strain gauge methods, numerical studies by finite element method and Koiter’s asymptotic analysis. Strain gauge methods for testing columns under compression were also used by Ye. et al. [2] and Ziółkowski and Imiełowski [3]. Zhou and Chen [4] investigated the compressive strength of channel columns with a partially closed cross-section. They performed experimental tests, using strain gauges and deflection sensors, and finite element analysis. Nowadays, as technology develops, other methods are also used, such as modern optical methods. Urbaniak et al. [5] analysed the effect of different boundary conditions on the critical force and on the force at failure of a composite column. The columns were loaded with a compressive force. For the study, they used classical strain gauge methods and modern optical methods such as the ARAMIS system. Optical methods have a lot of advantages over the strain gauge methods, and they provide insight into the displacement of entire columns. Strain gauge methods allow for the measurement of deformations only at points where strain gauges are applied. Optical methods have been used to study columns with channel cross sections by Czapski and Kubiak [6], Styles et al. [7] and Perret et al. [8]. Paszkiewicz and Kubiak [9] considered the issues of determining the buckling load in composite channel beams under bending and channel columns under compression. The data necessary to determine the load were collected using the strain gauge method and the optical method. The buckling load was determined using several methods, including the strain-averaged method. Kubiak et al. [10] in their paper presented optical testing of beams subjected to pure bending. They studied specimens with a pre-damaged web and flange in order to check the effect of impact damage on the performance of a thin-walled structure. Zhang et al. [11] performed an experimental and numerical study of the behaviour of circular thin-walled sections, made by additive methods, subjected to compression. Viguie and Dumont [12] analysed the buckling of box plates loaded with a compressive force. They used Digital Image Correlation (DIC) for the study. Paper [13] presents a technique, based on the use of topographic data obtained from optical measurements, for creating finite meshes representative of the geometry of real structures that can be analysed to obtain accurate predictions of unstable behaviour. Fortan and Rossi [14] used the DIC system to measure imperfections and displacements in beams subjected to four-point bending.

The most commonly used numerical methods in the study of thin-walled structures are the finite element method (FEM) and the finite strip method (FSM). The authors used the finite strip method for the study. The buckling analysis using a generalized complex finite strip method was performed by Naderian and Ronagh [15]. In a paper [16], Anbarasu discussed the strength, post-buckling behaviour and design of cold-formed channel beams. The properties studied were influenced by the interaction between the buckling forms: local and distortional. The study was performed using the finite strip method and Generalized Beam Theory (GBT). The purpose of the finite element research described in Czapski and Kubiak [17] is to investigate the effect of residual stresses on the behaviour of thin-walled laminates loaded in compression. Szewczak et al. [18] conducted a numerical analysis of simply supported beams. The beams were reinforced with special CFRP tapes. They were subjected to four-point bending. In the work [19], they extended the research, and optical tests were performed using the ARAMIS system. Zaczynska and Kazmierczyk [20] performed a parametric study of medium-length beams made of variable functional gradation materials (FGM) consisting of aluminium and titanium layers. In publication [21], the compression resistance, load-axial shortening response and deformed shapes are presented. Nonlinear finite element models were developed. The FE and the experimental results were found to be in good agreement in terms of ultimate compression resistance, deformed shapes and load-axial shortening curves.

The strength and stability of thin-walled sections is significantly influenced by the shape of the cross-section and, consequently, its geometrical characteristics. Scientists analyse and study more and more interesting shapes of cross-sections. The paper analyses selected cross-section shapes. Their choice was dictated by the desire to show the influence of individual modifications on the values of maximum forces, critical forces and buckling forms. The common part for all columns, in this case the box flange, was retained. The analysed cross-sections are manufactured by a Polish company. The conducted research has an implementation potential. The following papers describe tests with sections with modified shapes of cross-sections. It was noted that such complicated shapes as those analysed in this work have not been developed in other research units. Paczos [22] experimentally investigated beams with modified shapes of flanges. He investigated beams with double steel sheets on flanges formed into box and drop shapes. Magnucka-Blandzi and Magnucki [23] included modifications of the cross-section in the form of folds in their research, but also studied a beam having a sandwich structure on the flange. The problem of stability of a channel beam with an orthorthopic shape of the flange was handled by Magnucka-Blandzi and Zając [24]. Grenda and Paczos [25] investigated two channel sections having stiffeners on the flange and a modified web shape; the beams were subjected to pure bending. Huang et al. [26] investigated a channel section with edge bending; they applied an additional stiffener in the form of internal ribbing along the web. Naser and Degtyareva [27], El-Dehemy [28] and Kulatunga and Macdonald [29] investigated channels having different types of perforations on the web. Different shapes of perforations and different variants of their arrangement on the web were considered. Misiunaite et al. [30] performed experimental and numerical studies on beams made of high-strength steel. The study showed that the shape of the cross-section influences the efficiency of the use of high-strength steel.

## 2. Geometry of Column Cross-Sections and Material Properties

**Figure 1:**
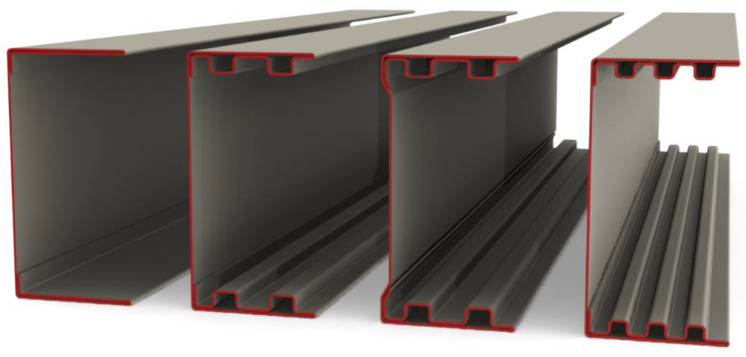
Shapes of cross-sections of tested columns.

The subject of the research are four thin-walled channel columns with modified cross-section shapes. The columns were made in the cold forming technology by a Polish company “Blachy Pruszyński”. The shapes of the analysed cross-sections are shown in Figure 1. The modification of the shape of the column cross-sections is aimed at assessing the influence of this modification on the strength, bearing capacity and resistance to loss of stability of the tested columns. The investigated columns had double metal sheets on the flange. The shape of the flange and the web was modified. Overall dimensions of all tested cross-sections are: height H = 80 mm, width b = 40 mm. The total length of columns tested was up to 1000 mm.

**Figure 2:**
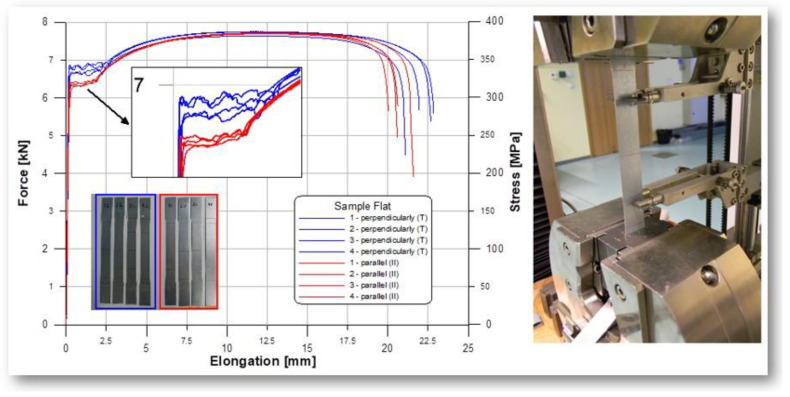
Research on the durability properties of DX51.

Columns were made of DX51 steel sheets with a wall thickness of t=0.5 mm and a hot-dip galvanized zinc coating $Z200-220\;g/m^{2}\;$(Z200 wg EN 10025-2:2007). The columns are made without disconnected and non-disconnected joints. The sheets on the flanges are not connected to each other. Material properties were measured on specimens cut from the columns. They were determined by testing five specimens prepared according to Eurocode 3. The results were obtained from the tensile test described in Paczos’ work [31] and are shown in Table 1 in Figure 2.

The above values of material properties were implemented into numerical tests, carried out using the finite strip method. Table 2 shows the detailed dimensions of the cross-sections of the tested columns and the position of the centres of gravity.

Geometrical characteristics of the cross-sections of the tested columns are presented in Table 3. The cross-sectional area A was determined, $z_{c}$ and $y_{c}$—coordinates of the position of the centre of gravity of the cross-section, $I_{\mathrm{xx}}$—axial moment about the *x*-axis, $I_{\mathrm{yy}}$—axial moment about the y-axis and $I_{\mathrm{zy}}$—deviation moment.

The main purpose of determining the geometrical characteristics was to determine the position of the centre of gravity of the tested columns, as these data were necessary to determine the point at which the compressive force should be applied.

## 3. Experimental Investigation

Experimental tests were carried out using two methods: the classical strain gauge method and the modern optical method. On the basis of strain gauge tests, the values of critical forces were determined. The optical method was used to determine the buckling force and buckling forms of the columns. The buckling force was defined as the force at which the first signs of buckling appeared.

### 3.1. Test Stand

**Figure 3:**
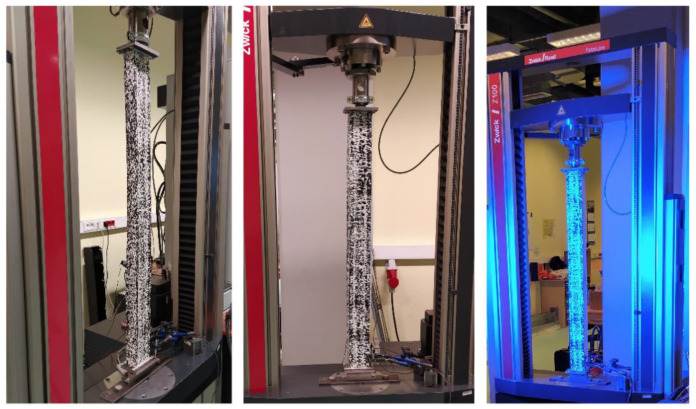
Test columns mounted in the testing machine.

The tests were carried out using a ZWICK Z100/Roell testing machine. This is a universal machine that can perform compression, tension and bending tests of 0.2–100 kN. The specimens were loaded with a progressively increasing axial compressive force applied at the geometric centre of gravity of the cross-section of the individual columns. The conducted research belongs to the group of static research. An initial force of 50 N was applied to the columns. Then the force was gradually, statically increased at a rate of 5 mm/min. This applied load resulted in conditions corresponding to pure compression. Special spacers were used to prevent uncontrolled movement of the column ends during application of the force. The upper spacer, to which the compressive force was applied, blocked two translations with respect to the *y*-axis and the *z*-axis. Translation relative to the *x*-axis was possible. Three rotations with respect to all axes were also blocked. The bottom spacer blocked three translations and three rotations. The coordinate system used is shown in Figure 2. Owing to the chosen boundary conditions, free support was obtained so that the system was not stiffened. Figure 3 shows the tested columns mounted in the testing machine.

**Figure 4:**
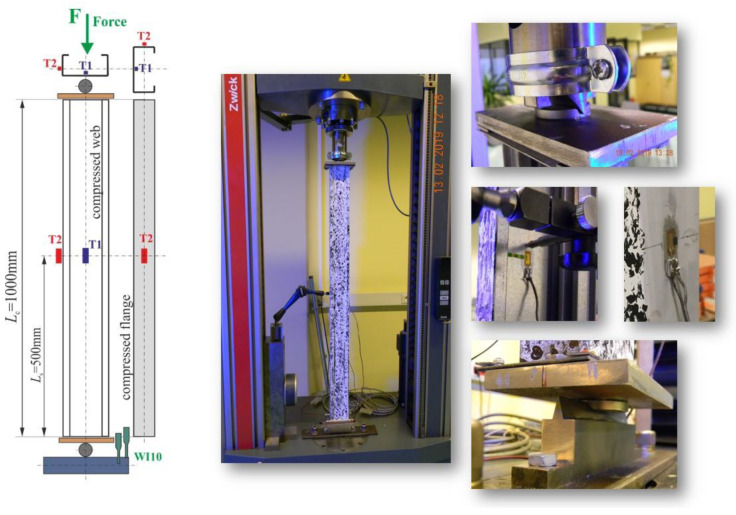
Test stand.

The test stand is shown in Figure 4. The tests were carried out until the columns failed, which means until their load capacity was completely exhausted. Strain was recorded using the strain gauge method and displacement was recorded using the optical method at the same time. In addition, both strain gauges and optical systems recorded the compressive force. The tests were carried out in accordance with the guidelines of Eurocode 3.

### 3.2. Strain Gauge Tests

Classical strain gauge tests were performed. Two strain gauges were glued on each of the tested columns. The first one (T1) was glued on the web in the middle of the column height, the second one (T2) was glued on the flange in the middle of the column height. The location of the strain gauges is shown in Figure 4. The points at which the strain gauges were glued are due to the fact that the largest deformations occur at these points. Linear foil resistance strain gauges 1-LY11-6/350 manufactured by HBM company, with the size of the resistance part of 2.8 × 6 mm and a strain gauge constant equal to K = 2.1, were used in the tests. During the measurement, the following were recorded: measurement time, compressive force, strain and shortening of the columns. The recording was performed using a computer method.

**Figure 5:**
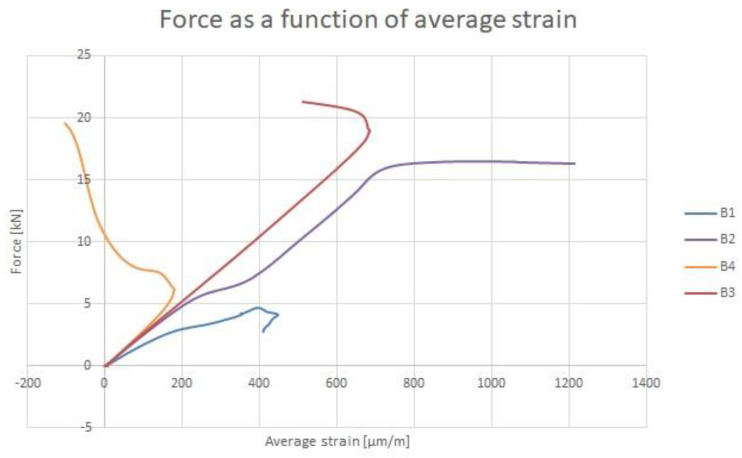
Force as a function of strain averaging. Results of strain gauge testing.

The critical force and the maximum force were determined from strain gauges. The maximum force was defined as the force at which failure of the column occurred. The critical force values were determined using the average strain method and the method in which the critical force value was obtained from the point where the regression line starting at the origin of the coordinate system ceases to coincide with the rectilinear section of the diagram. Using the first method, the critical force value was obtained for columns B1, B2 and B4. The second method was used to determine the value of the critical force for column B3. The use of two different methods was not accidental. The averaged strain method involves creating a plot of compressive force versus averaged strain and dividing this plot into pre-buckling and post-buckling states. The average strain is the arithmetic mean calculated from the strains of the two strain gauges. The pre-buckling and post-buckling states, represented as parts of the graph, are approximated linearly and the intersection of these lines is found. The point obtained (its y-coordinate) indicates the value of the critical force. In the case of column B3, it was not possible to divide the graph into the pre-buckling and post-buckling states, due to the course of this graph. The methods used are described in papers [32,33]. The graphs of compressive force as a function of averaged strain are shown in Figure 5.

**Figure 6:**
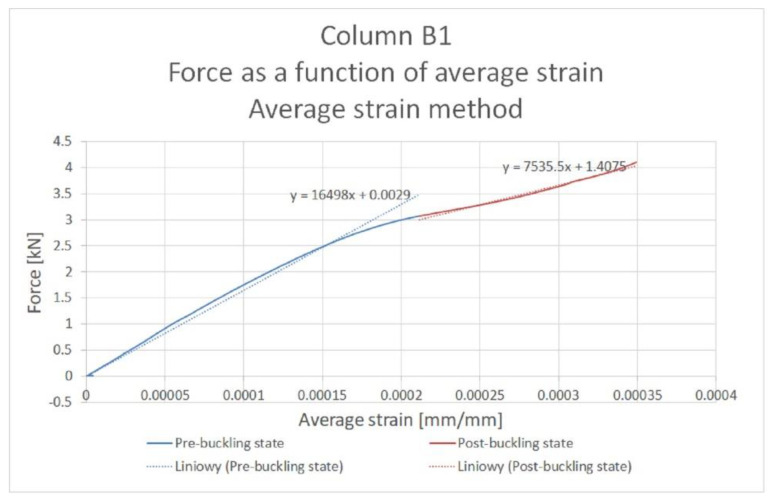
Procedure for determining the critical force by the strain average method using the example of column B1.

The procedure for determining the critical force value, using the averaged strain method, is presented using column B1 as an example. Figure 6 shows the division of the strain averaged as a function of compressive force into pre-buckling and post-buckling states. The pre-buckling state is defined as that part of the curve which is linear up to the inflection point. Once the inflection point is exceeded, the post-buckling state begins.

Next, regression lines were determined for both states. The equations of these lines were determined, visible in Figure 5. Next, the point of intersection of the regression lines was determined. This is the point with coordinates (162.78; 2.68). Thus, the value of the critical force for column B1 is 2.68 kN. In order to determine the values of critical forces for columns B2 and B3, an analogous procedure to that implemented for column B1 was used.

**Figure 7:**
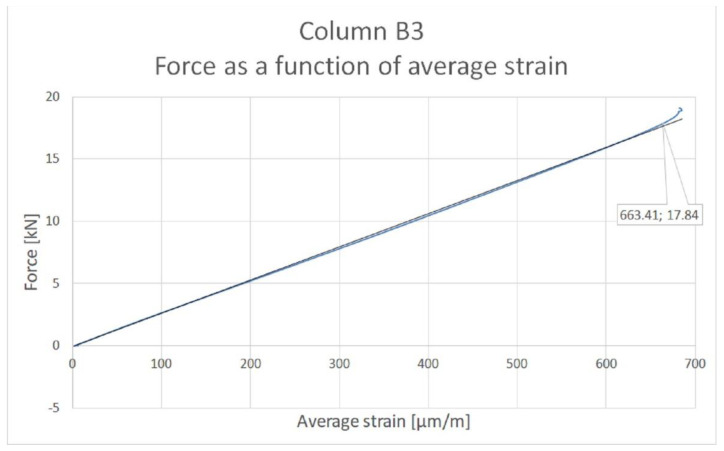
Determination of critical force value for column B3.

The procedure for determining the critical force for column B3 is shown in Figure 7. The plot of mean strain versus compressive force was approximated by a linear regression starting at the origin of the coordinate system.

The point at which the graph ceases to coincide with the regression line was then determined. Based on this method, the point with coordinates (663.4; 17.8) was determined. Thus, the critical force value for column B3 is 17.8 kN. The values of critical forces and maximum forces are shown in Table 4.

The highest value of the critical force was obtained for column B3 and the lowest for column B1. For the maximum forces, the situation is analogous, with the highest value obtained for column B3 and the lowest for column B1. Column B1 has only a double plate on the flange and an internal bend. On the other hand, column B3 has a double plate on the flange, but also a non-standard shape of the flange and the web.

### 3.3. Optical Tests

Tests were carried out using the ARAMIS system, which allows the measurement of deformations of the structure in real time. Sensors are used to measure 3D dynamic coordinates, 3D displacements and 3D displacement deformations. ARAMIS uses a triangulation system for the measurements: a method of measuring distances using an optical rangefinder which consists of a light source (transmitter) and a receiver (lens) with a known position and unequal optical axes. The columns were coated with paint so that a black and white pattern was created on them. A 3D optical scanner detects the points formed on the columns by using two contrasting colours. The optical system used takes photographs which, when assembled, form a film. It is possible to analyse the deformations frame by frame. The images taken were analysed using the GOM Correlate software. This software allows one to check the deformation values at specific points. It is also possible to check the highest or lowest strain value in a given area of the column. In addition, the strain and displacement values are shown as a colour gradient. This allows us to observe and easily recognise the forms of column buckling (local, general and distortional). Figure 1 indicates the coordinate system that was implemented for all the columns under study. The coordinate system used is the same as that used for the strain gauge method for determining the position of the centre of gravity and for the finite strip method.

The buckling force at which the first signs of buckling appeared, the maximum force and the buckling form of the columns were determined from the optical tests. The results obtained are presented in Table 5.

**Figure 8:**
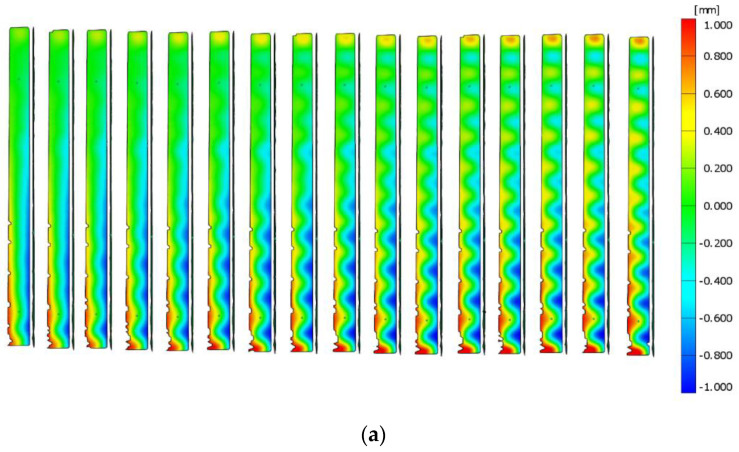

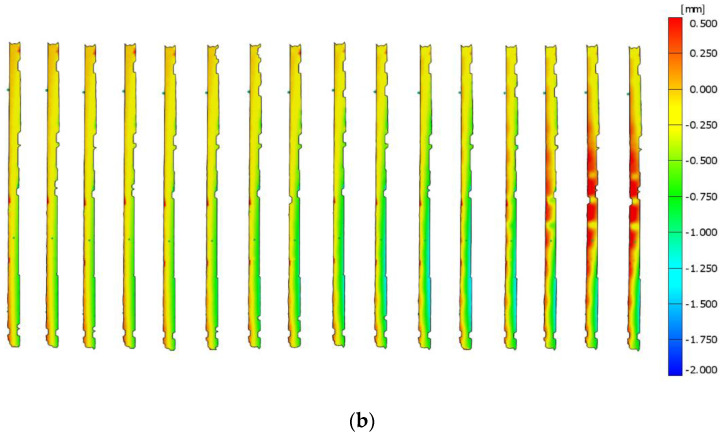
(**a**) ARAMIS test results for column B4—web displacement. (**b**) ARAMIS test results for column B4—flange displacement.

Figure 8 shows the displacements for column B4 obtained from the optical tests. The deformations of the webs and flanges are visible. By using an appropriate scale, it is possible to determine at which points the maximum deformations occurred. In the case of each column, the largest deformations occurred in the middle of the column length.

In case of column B1, the loss of stability occurred through distortional–local buckling. In the case of the other columns, half-waves, characteristic for local buckling, were formed.

**Figure 9:**
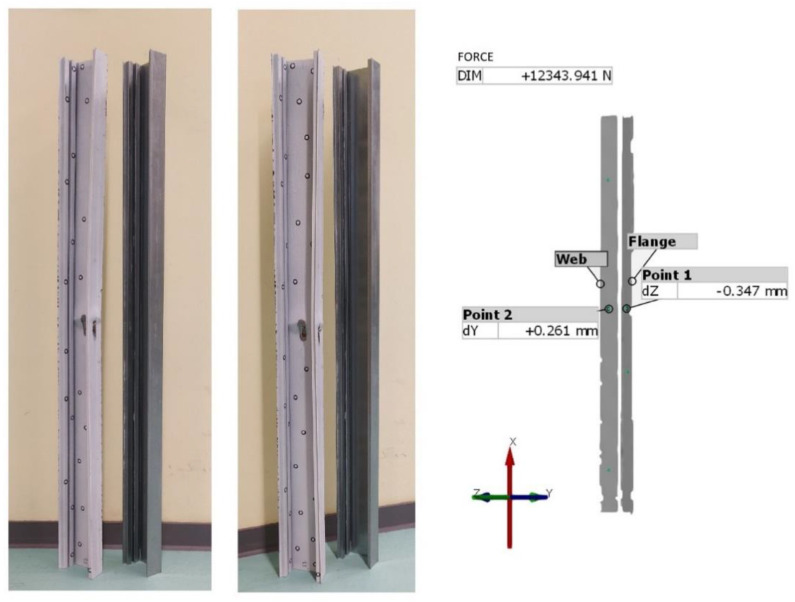
Location of measurement points on the surface of the tested column. GOM Correlate program.

In addition, the GOM Correlate program allows the measurement of displacements, at specific points in the columns. Two measurement points were generated for each column. The location of these points was the same as the location of the strain gauges. Table 6 shows the displacement values for these points when the columns were loaded with the critical force and the maximum force. On the other hand, Figure 9 shows the locations of the measuring points, using column B4 as an example.

The largest displacements, as per absolute value, at the critical force and maximum force were obtained for column B1, i.e., for the column with the least complicated cross-sectional shape. In the case of columns with a more complicated cross-section, the values of displacements, as per absolute value, at the critical force and maximum force are similar.

**Figure 10:**
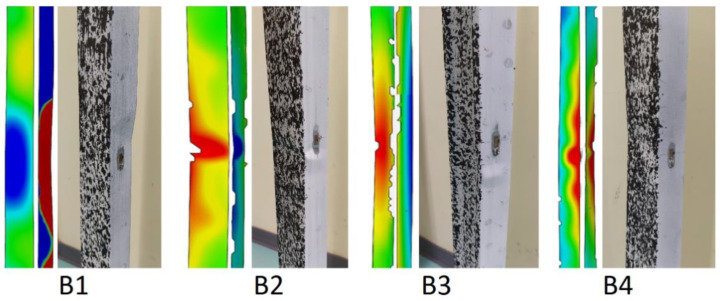
Forms of destruction of columns.

By using optical methods, it is also possible to observe the forms and course of column destruction, which are shown in Figure 10. By using a colour gradient as a displacement map, it is apparent where column destruction has occurred.

The forms of destruction of all the columns are similar. The destruction of all columns occurred in the middle of their length.

## 4. Numerical Analysis Using the Finite Strip Method

Numerical investigations using the finite strip method were carried out using the CUFSM v5.04 program. The finite strip method is derived from the finite element method in terms of its calculation method (partitioning of an element into strips, shape function); thus, the accuracy of this method depends on the partitioning of the element into stripes. On the basis of the tests with the finite strip method, the values of critical forces and the percentage contribution of individual forms of buckling to the loss of stability of the tested columns were determined. This method also makes it possible to observe the deformation of the column during the application of a gradually increasing compressive force.

Finite strip testing is carried out, in the first instance, by providing the input data, i.e., the material properties and the cross-sectional shape of the column. The actual values of material properties and cross-section dimensions are implemented in the program. Then, by using the load generator, the load was given; in the case of the described research, it was a gradually increasing compressive force applied at the centre of gravity of the cross-section. In the next step, the boundary conditions (free support) were given. The application of the described loads and boundary conditions caused the tests to be carried out in accordance with the guidelines contained in Eurocode 3, so that it was possible to verify these results by comparing them with the results obtained from experimental methods.

**Figure 11:**
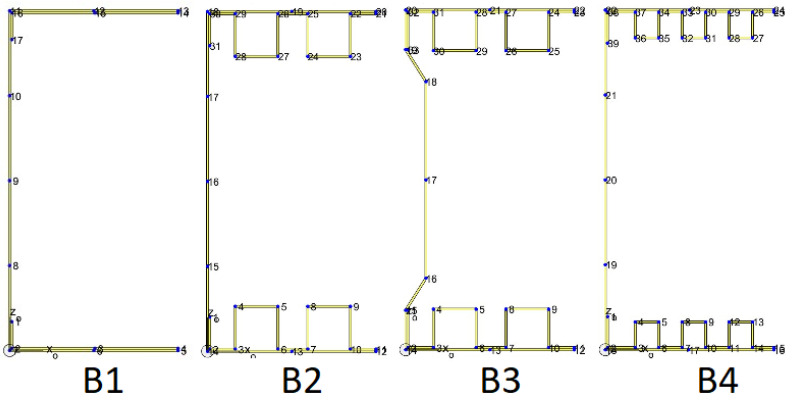
Division of the analysed cross-sections into strips.

In the subsequent steps of the finite element method, the cross-section must be divided into stripes; in the case of the analysed columns, the stripes are shown in Figure 11. Each wall, including the walls of the box girders and end bends, is treated as a separate strip.

Column B1 was divided into 16 strips, column B2 into 30 strips, column B3 into 32 strips. Column B4 has 38 strips. The stripes are of different lengths. In the case of modified flange shapes, the length of each flange is equal to the length of the box sides. However, the outer sheet on the flanges is divided into two strips, and the sheet on the webs into four strips, except for column B8, which has a non-standard web shape. For column B3, the web is divided into six strips. For column B2, a check was made on the influence of the number of finite stripes on the results obtained. The analysis has shown that the optimum number of stripes on the web is four, and on the flanges two. The results of the analysis are presented in Table 7.

**Figure 12:**
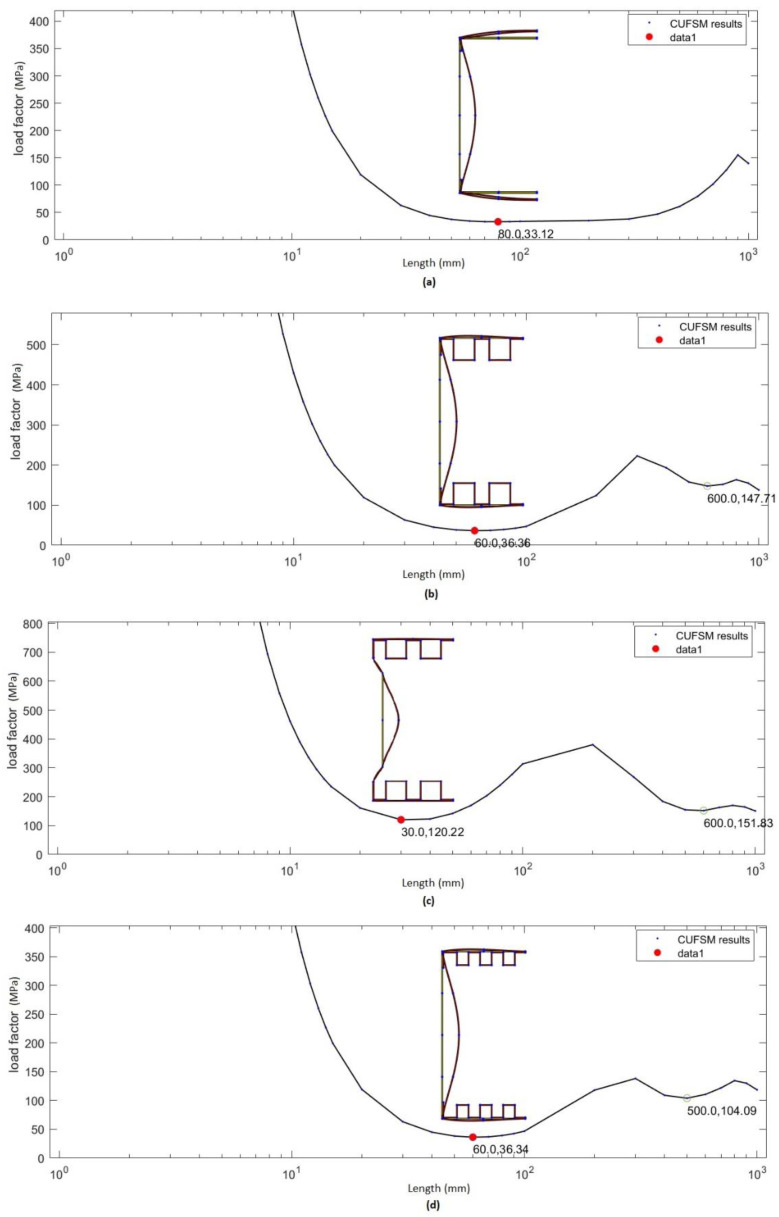
Results of the simulation carried out using the finite strip method: (**a**) Results for column B1. (**b**) Results for column B2. (**c**) Results for column B3. (**d**) Results for column B4.

Figure 12 shows the results obtained from the finite strip method. Load factor corresponds to the value of critical stress. Knowing the value of the cross-sectional area of the columns, it is not difficult to determine the value of the critical force for the columns analysed.

The values of stresses and critical forces obtained from the finite strip tests are shown in Table 8.

**Figure 13:**
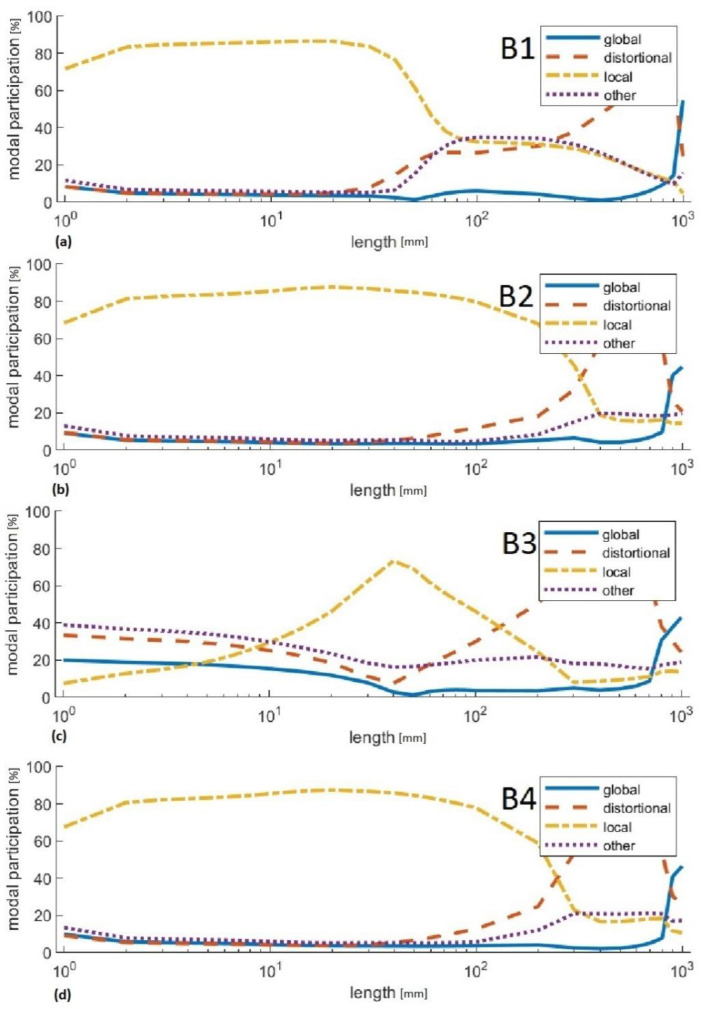
Percentage contribution of individual buckling forms to the loss of stability of the tested columns: (**a**) Results for column B1. (**b**) Results for column B2. (**c**) Results for column B3. (**d**) Results for column B4.

The percentages of the respective buckling forms in the loss of stability of the analysed columns were also determined. The results are presented in Table 9 and Figure 13.

The highest value of critical force was obtained for column B3, and the lowest for column B1. In the case of the maximum force, the highest value was obtained for column B3, and the lowest for column B1. The percentages of the different forms of buckling showed that columns B2, B3 and B4 are subjected to local loss of stability. Column B1 is subject to distortional–local loss of stability, but it should be noted that the contribution of the other form of buckling is as high as 33%.

## 5. Discussion of the Results

Four thin-walled channel columns with non-standard cross-section shapes were tested experimentally and numerically. Tests were carried out in accordance with procedures included in Eurocode 3. Geometrical characteristics of cross-sections were determined. The aim of the research was to determine which cross-section has the most favourable geometrical characteristics under the influence of axial compressive force applied at the centre of gravity of the cross-section of tested columns.

Experimental investigations were carried out using two methods: the strain gauge method and the digital image correlation method, with the use of a modern ARAMIS system. With the use of strain gauges, the value of critical force, at which the loss of stability of columns occurs, and the value of maximum force, at which the total loss of load-bearing capacity occurs, were determined. In the case of optical tests, the buckling force at which the first signs of buckling occur, the maximum force and the buckling form of the columns were determined. On the other hand, numerical investigations were carried out using the finite strip method. Owing to this method, the values of critical forces and the percentage shares of particular buckling forms in the loss of stability of the tested columns were determined.

**Figure 14:**
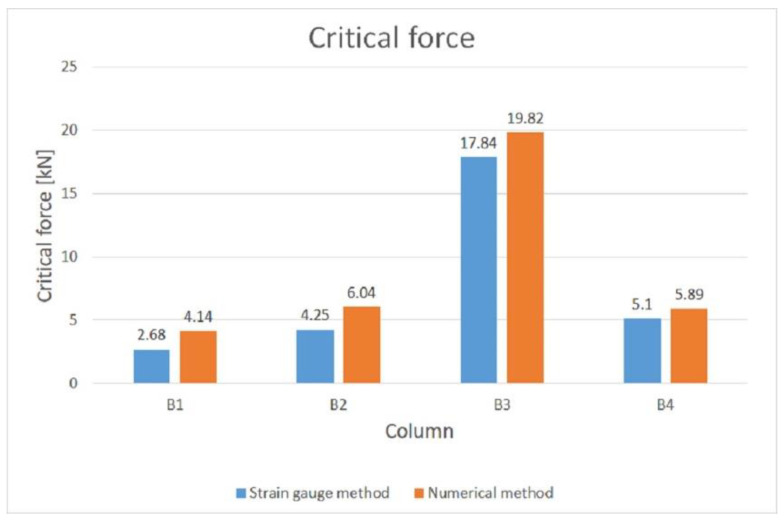
Comparison of critical force values obtained from strain gauge and numerical CuFSM methods.

A comparison of the critical forces obtained from the strain gauge method and the numerical method is shown in Figure 14. The highest value of the critical force was obtained for column B3. Based on the performed tests, the buckling forms for the analysed columns were unambiguously determined. Column B1 undergoes distortional–local loss of stability, while columns B2, B3 and B4 undergo local loss of stability. In the case of column B1, the value of critical force obtained from the strain gauge method is 35% lower than the critical force obtained for this column from the numerical method. For column B2 this difference is 30%. For column B3 it is 10%, and for column B4 13%.

**Figure 15:**
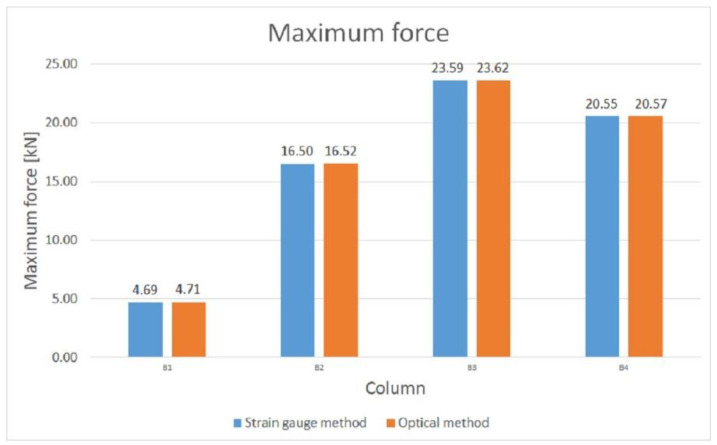
Comparison of maximum forces obtained using strain gauge and optical methods.

A comparison of the values of the maximum forces obtained from the strain gauge and optical tests is shown in Figure 15. Based on the analysis of the tests carried out, it was determined that column B3 has the highest load capacity. The differences between the values of the maximum forces obtained from the two methods are very small. The failure mechanisms for all columns are similar, with failure occurring in the middle of the column length.

An important aspect in evaluating the columns analyzed is their weight. Table 10 presents a summary of the weights of the individual columns along with the load-carrying capacities and critical forces obtained for them.

The weight of column B2 increases by 10% over that of column B1. With this small increase in weight, the critical force grows by 56%, and the load carrying capacity increases by 3.5 times. When comparing column B3 and column B1, the largest increases in critical force and maximum force values were observed. With only a 30% weight growth, the critical force increases by more than six times, while the load capacity increases by 3.8 times. Column B4 in its cross section has three bends with lower height compared to the other columns with modified flange shape. The weight of the B4 columns, compared to the B1 column, increases by 10%. For this pair of columns, there is a 90% increase in the critical force value in favour of the column with the modified cross-sectional shape. The load capacity of column B4 with respect to column B1 increases by as much as 438%. For columns B2 and B4, the weight increase, relative to column B1, is the same at 10%. However, in terms of critical force value and load carrying capacity, column B4 is much more favourable. The significant influence of the cross-sectional shape on strength and resistance to loss of stability can be seen.

**Figure 16:**
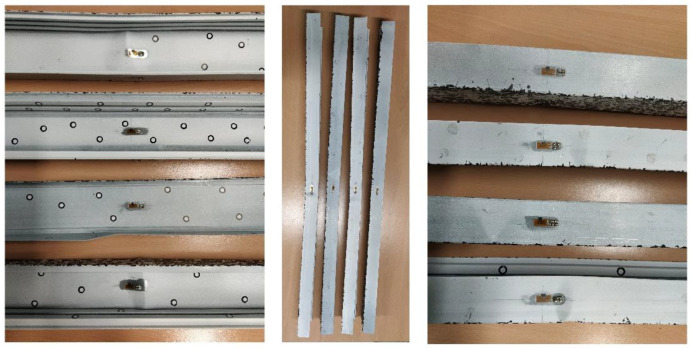
Mechanisms of failure of tested columns.

All the columns analysed collapsed around the middle of the column height. The failure mechanisms for all columns are similar and are shown in Figure 16.

**Figure 17:**
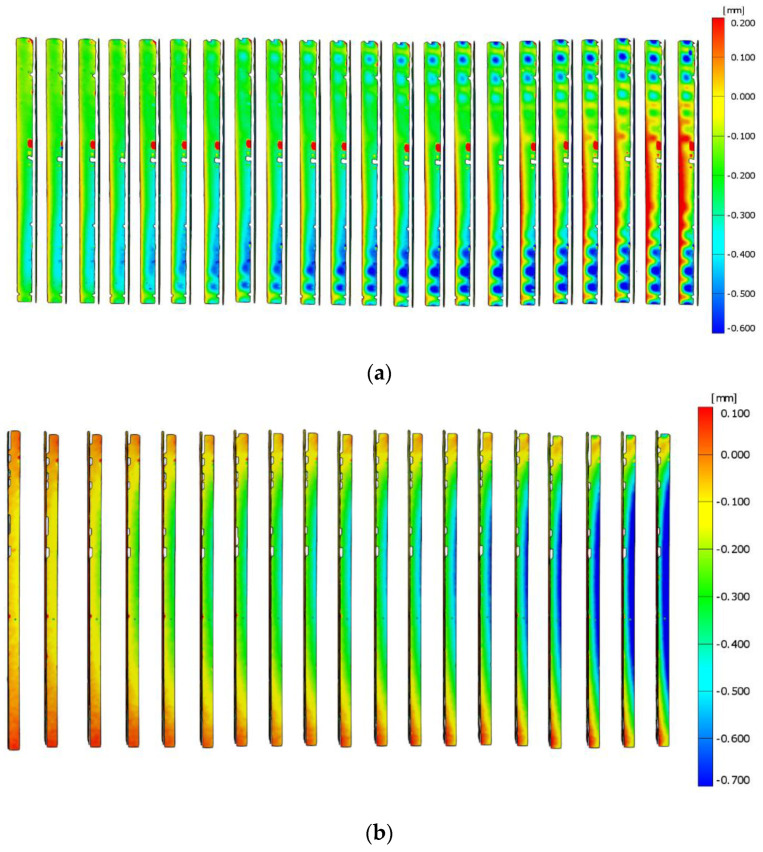
(**a**)**.** Results of tests carried out with ARAMIS for column B3—displacements on web. (**b**) Results of tests carried out with ARAMIS for column B3—displacements on flange.

The most favourable cross-section in terms of axial compressive force is that of column B3. The web and flange displacements, obtained from the optical method, for this column are shown in Figure 17.

## 6. Conclusions

Column B3 has the most favourable geometrical characteristics in terms of axial compressive force. The critical force and the maximum force are the highest for this column. The characteristic feature of this column, compared to the others, is that it has a modified web shape. Column B2 also deserves special attention because the shape of the flange in this column is the same as in column B3, but it does not have folds on the web. It is observed that the critical force value for column B2 is four times lower than for column B3. However, the maximum force for column B2 is 7.09 kN lower than that for column B3.

Columns B2 and B4 have a modified flange shape: column B2 has two boxes on the flange, while column B4 has three boxes. In the case of column B4, the boxes are smaller in size than in column B2. The critical and maximum forces for column B4 reach higher values than those for column B2. The maximum force for column B4 is 4.05 kN higher than that for column B2. For the critical force, this difference is only 0.85 kN.

## Figures and Tables

**Table 1:** Strength properties of DX51.

Properties	Symbol	Value
Young’s modulus	E	181 GPa
Tensile yield strength	$R_{\mathrm{eH}}$	330 MPa
Tensile strength	$R_{m}$	380 MPa

**Table 2:** Cross-sectional dimensions of tested columns.

**B1**	**B2**
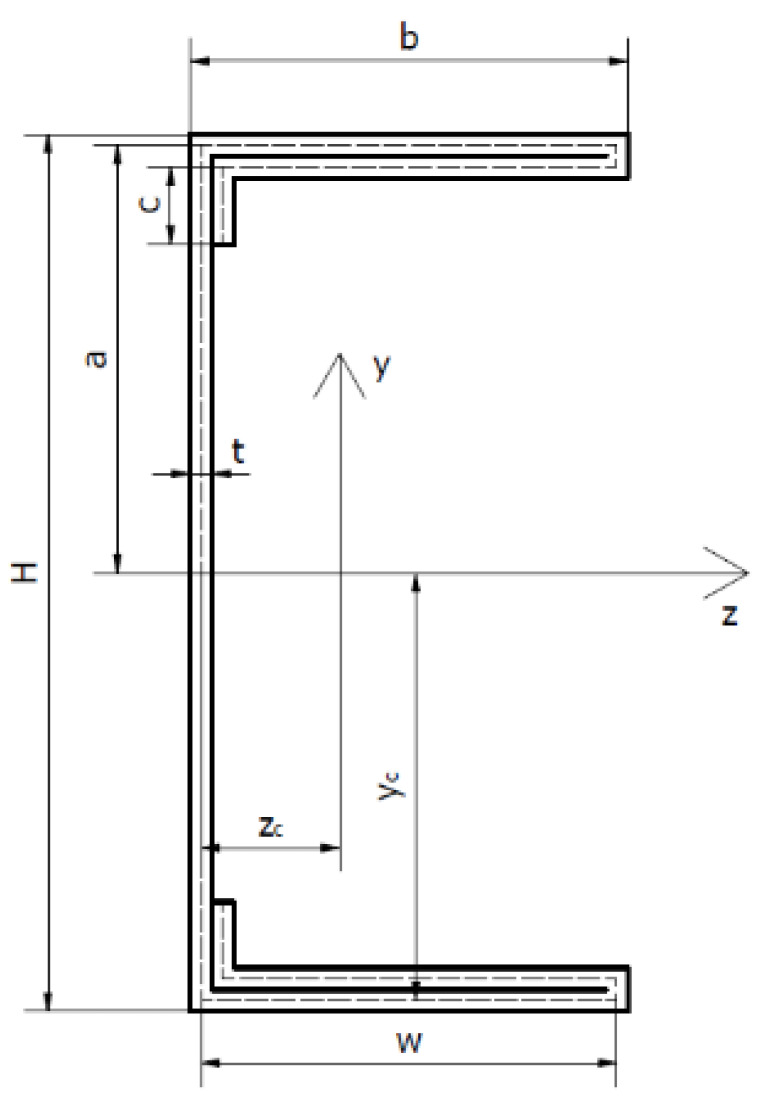	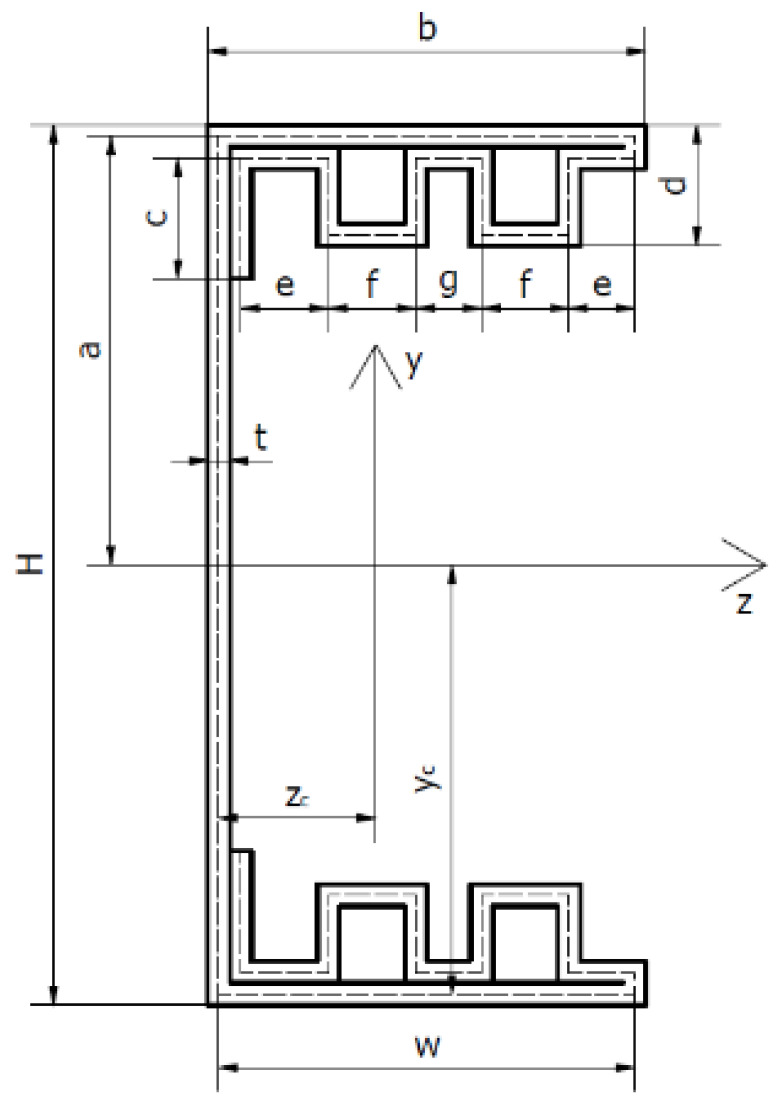
t=0.50 mm H=80.00 mm b=40.00 mm a=39.75 mm w=39.5 mm c=6.25 mm	t=0.50 mm H=80.00 mm b=40.00 mm a=39.75 mm w=39.50 mm d=11.00 mm c=7.50 mm f=10.00 mm g=7.00 mm e=6.00 mm
**B3**	**B4**
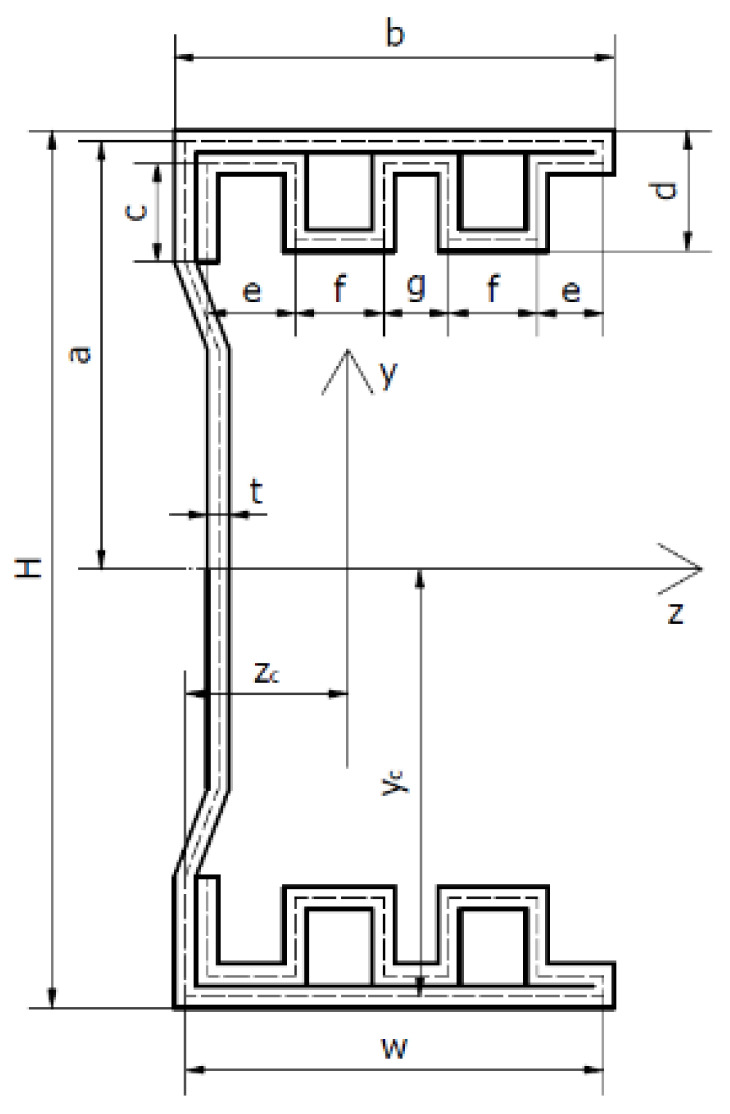	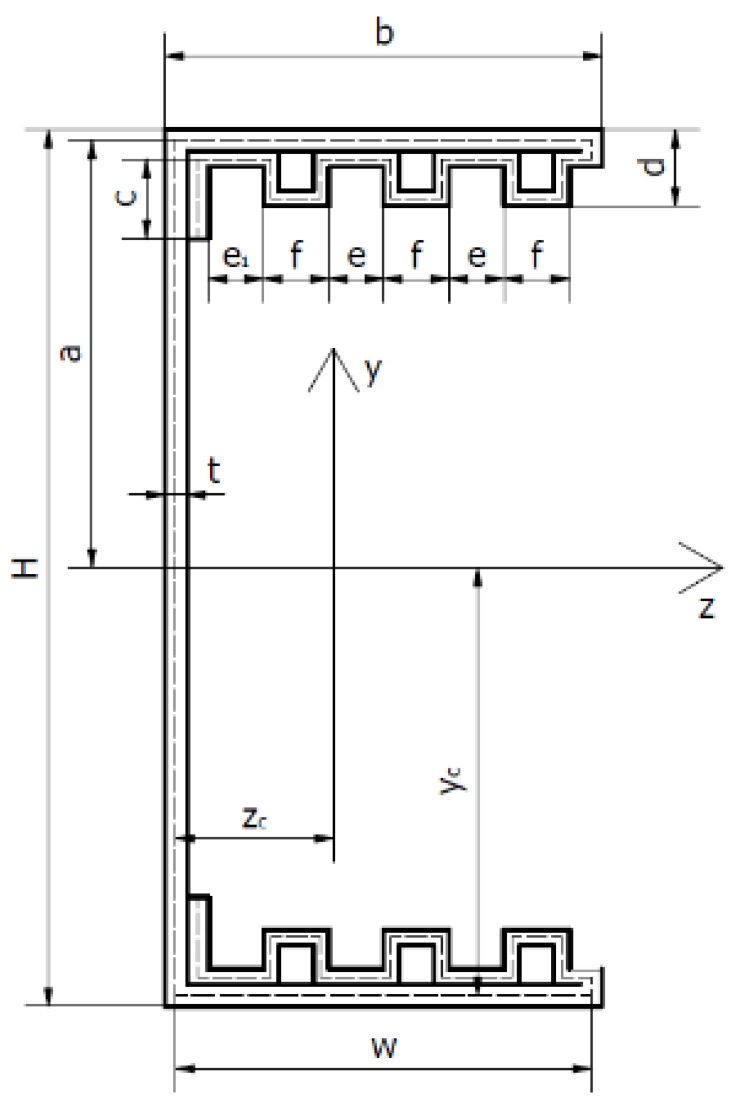
t=0.50 mm H=80.00 mm b=40.00 mm a=39.75 mm w=39.50 mm d=10.00 mm c=8.75 mm k=4.50 mm f=10.00 mm g=7.00 mm e=6.00 mm	t=0.50 mm H=80.00 mm b=40.00 mm a=39.75 mm w=39.50 mm d=7.00 mm e=5.00 mm $e_{1}=6.00\;\mathrm{mm}$

**Table 3:** Geometric characteristics of cross-sections

Columns	$A\;\left(mm^{2}\right)$	$z_{c}\;\left(mm\right)$	$y_{c}\;\left(mm\right)$	$I_{xx}\;\left(mm^{4}\right)$	$I_{yy}\;\left(mm^{4}\right)$	$I_{zy\;}\left(mm^{4}\right)$
B1	125.00	12.66	39.75	152,387.06	21,821.12	0.00
B2	166.25	14.34	39.75	187,253.23	27,583.09	0.00
B3	164.88	14.77	39.75	187,106.02	24,406.47	0.00
B4	162	14.39	39.75	193,680.00	27,018.87	0.00

**Table 4:** Values of critical and maximum forces determined from strain gauges.

Column	Critical Force [kN]	Maximum Force [kN]
B1	2.68	4.69
B2	4.25	16.50
B3	17.84	23.59
B4	5.10	20.55

**Table 5:** Values of buckling forces, maximum forces and buckling modes of columns obtained from the optical method.

Column	Buckling Force [kN]	Maximum Force [kN]	Form of Buckling
B1	1.68	4.71	local–distortional
B2	3.82	16.52	local
B3	8.49	23.62	local
B4	5.03	20.57	local

**Table 6:** Displacement values for flange and web measurement points obtained using the optical method.

Column	Displacement at Critical Force on Web [mm]	Displacement at Critical Force on Flange [mm]	Displacement at Maximum Force on Web [mm]	Displacement at Maximum Force on Flange [mm]
B1	0.258	−0.815	−5.005	−4.755
B2	−0.073	−0.066	3.473	3.538
B3	0.125	0.122	3.291	3.512
B4	−0.159	−0.182	3.641	3.328

**Table 7:** Analysis of the influence of the number of stripes on the flanges and on the web of the numerical analysis results of CuFSM.

Number of Strips on the Flanges	Number of Strips on the Web	Load Factor
1	1	48.34
2	2	36.79
2	4	36.36
4	8	36.32
8	16	36.31

**Table 8:** Values of critical stresses and critical forces obtained from numerical simulations performed with the finite strip method.

Column	Critical Stress [MPa]	Critical Force [kN]
B1	33.12	4.14
B2	36.36	6.04
B3	120.22	19.82
B4	36.34	5.89

**Table 9:** Percentage contribution of individual buckling forms to the loss of stability of the tested columns.

Column	Global Buckling	Distortional Buckling	Local Buckling	Other Form of Buckling
B1	5.5%	26.8%	34.7%	33.0%
B2	3.4%	7.7%	83.9%	4.9%
B3	7.9%	11.2%	62.4%	18.5%
B4	3.5%	8.1%	83.2%	5.2%

**Table 10:** Comparison of column weights with values of resistance and critical forces obtained from strain gauge tests.

Column	Weight [kg]	Maximum Force [kN]	Critical Force [kN]	F_CR_/F_MAX_
B1	0.861	4.69	2.68	0.57
B2	0.947	16.5	4.25	0.26
B3	1.115	17.84	23.59	0.76
B4	0.948	20.55	5.1	0.25
B1/B1	1.00	1.00	1.00	1.00
B2/B1	1.10	1.56	3.52	0.44
B3/B1	1.30	6.66	8.80	0.75
B4/B1	1.10	1.90	4.38	0.43

## Data Availability

Not applicable.
